# Screening for latent TB, HIV, and hepatitis B/C in new migrants in a high prevalence area of London, UK: a cross-sectional study

**DOI:** 10.1186/s12879-014-0657-2

**Published:** 2014-12-03

**Authors:** Sally Hargreaves, Farah Seedat, Josip Car, Rod Escombe, Samia Hasan, Joseph Eliahoo, Jon S Friedland

**Affiliations:** Imperial College London, Department of Medicine, Section of Infectious Diseases and Immunity, Hammersmith Hospital Campus, 8th Floor Commonwealth Building, DuCane Road, London, W12 ONN UK; Hammersmith and Fulham Centres for Health, Hammersmith Hospital, Hammersmith, London,

**Keywords:** Migrants, Hepatitis, Tuberculosis, HIV, Screening, Latent tuberculosis, Immigrants, Health service delivery

## Abstract

**Background:**

Rising rates of infectious diseases in international migrants has reignited the debate around screening. There have been calls to strengthen primary-care-based programmes, focusing on latent TB. We did a cross-sectional study of new migrants to test an innovative one-stop blood test approach to detect multiple infections at one appointment (HIV, latent tuberculosis, and hepatitis B/C) on registration with a General Practitioner (GP) in primary care.

**Methods:**

The study was done across two GP practices attached to hospital Accident and Emergency Departments (A&E) in a high migrant area of London for 6 months. Inclusion criteria were foreign-born individuals from a high TB prevalence country (>40 cases per 100,000) who have lived in the UK ≤ 10 years, and were over 18 years of age. All new migrants who attended a New Patient Health Check were screened for eligibility and offered the blood test. We followed routine care pathways for follow-up.

**Results:**

There were 1235 new registrations in 6 months. 453 attended their New Patient Health Check, of which 47 (10.4%) were identified as new migrants (age 32.11 years [range 18–72]; 22 different nationalities; time in UK 2.28 years [0–10]). 36 (76.6%) participated in the study. The intervention only increased the prevalence of diagnosed latent TB (18.18% [95% CI 6.98-35.46]; 181.8 cases per 1000). Ultimately 0 (0%) of 6 patients with latent TB went on to complete treatment (3 did not attend referral). No cases of HIV or hepatitis B/C were found. Foreign-born patients were under-represented at these practices in relation to 2011 Census data (Chi-square test −0.111 [95% CI −0.125 to −0.097]; p < 0.001).

**Conclusion:**

The one-stop approach was feasible in this context and acceptability was high. However, the number of presenting migrants was surprisingly low, reflecting the barriers to care that this group face on arrival, and none ultimately received treatment. The ongoing UK debate around immigration checks and charging in primary care for new migrants can only have negative implications for the promotion of screening in this group. Until GP registration is more actively promoted in new migrants, a better place to test this one-stop approach could be in A&E departments where migrants may present in larger numbers.

**Electronic supplementary material:**

The online version of this article (doi:10.1186/s12879-014-0657-2) contains supplementary material, which is available to authorized users.

## Background

International migrants have high levels of infectious disease, which has important implications for public health services [1],[2]. Approximately 70% of newly diagnosed UK tuberculosis (TB) cases London are in migrants - mainly as a result of reactivation of latent TB acquired some years earlier - with most cases of active disease presenting within 3–5 years of arrival [3],[4]. TB rates in the UK remain amongst the highest in western Europe [5]. In addition, around 60% of HIV cases are in migrants – mostly individuals from sub-Saharan Africa, of which late presentation to health services remains an important issue because of the barriers to health care they face [3]. Comparable trends are expected for both hepatitis B and C [3],[6]. How best to improve the delivery of cost-effective screening programmes for this often hard-to-reach group remains the subject of ongoing debate [7]-[9].

Screening approaches vary across UK and Europe [10], with emphasis placed on identifying and treating active TB on arrival in new entrants. In the UK, port of entry tuberculosis x-ray screening of new arrivals has recently been dismantled, amid calls to strengthen primary-care-based screening programmes and to take a more pro-active approach to screening in this group [3],[7],[8]. There is a renewed focus being placed on latent TB screening as a means of tackling high rates of active TB in the UK at the current time, with evidence of cost-effectiveness [11], yet there is considerable variability in approach to latent TB screening UK-wide, and deviations from national guidelines [9],[12]. Systematic screening for chronic hepatitis B infection among migrants is likely to be cost effective [13]. The National Institute for Health and Clinical Excellence (NICE) has issued guidance to ensure HIV testing is widely available in a range of healthcare and community settings, including General Practitioner (GP) practices [14]. Public Health England has recommended an “extended” New Patient Health Check at GP surgeries to explore broader health needs and to tackle the barriers to health care that newly arrived migrants face, as well as targeted TB screening and education [3],[15].

Whether GP practices offer a good opportunity to engage newly arrived migrants in screening initiatives remains unclear. In the UK, GPs act as gatekeepers to services available on the National Health Service (NHS) and so permanent registration with GPs on arrival to the UK is essential to facilitating access. On the one hand recent modelling work has shown that new migrants present a substantial burden on primary care in the UK [16], suggesting they present in large numbers, whereas other studies indicate otherwise [17],[18]. Migration status is not routinely recorded at UK health services, making data collection in this patient group challenging.

We undertook a cross-sectional study to test a new screening intervention that offered new migrants (in the UK ≤ 10 years) a one-stop blood test for HIV, latent TB, and hepatitis B and C on registration with a GP in primary care. Our aim was to investigate whether the intervention would increase the detection and treatment of these diseases and to explore feasibility and acceptability of this screening approach in the GP context.

## Methods

The study was carried out in 2013 for a 6 month period in two purposively selected primary care GP practices located adjacent to hospital Accident and Emergency Departments in a high migrant area of West London (Hammersmith and Fulham), where 42.8% residents are defined as foreign born/migrants according to the 2011 UK national population Census [19]. The survey site has the tenth highest level of foreign-born nationals of all local authorities in England and Wales, and is the eight highest in terms of the proportion of foreign-born people who have resided in the UK for less than 5 years [19].

Awareness-raising educational sessions were held for all GP and practice staff prior to the start of the study, and specific training carried out with the healthcare assistant and practice nurses on research processes (including participant recruitment and acquiring informed consent). All new patients registering at these two practices were given information about the study in 6 dominant local languages (Arabic, English, Farsi, Gujarati, Polish, and Somali) as part of their New Patient Registration Pack.

The existing procedure at these GP Practices, prior to our study commencing, was to offer all new patients who permanently register at either site a New Patient Health Check. The New Patient Health Check is carried out by the healthcare assistant or practice nurse once a patient has permanently registered and includes a general health check up and – more recently – an HIV test. There is no formal screening programme in place for latent TB, hepatitis B, and hepatitis C at these Practices; such testing is done at the individual GP’s discretion. Our aim, therefore, was to offer patients who met our inclusion criteria additional testing for latent TB, hepatitis B, and hepatitis C at the New Patient Health Check. We incorporated new questions into the template for the INPS-Vision computer system (a standard system used across UK primary care) to prompt staff to offer the one-stop blood test to eligible patients and facilitate routine data collection around migration status, an approach used successfully in a previous trial in the same context [20]. Inclusion criteria were: (a) foreign-born individuals who have lived in the UK ≤ 10 years who, prior to entry, had lived (≥1 years) in a country with a prevalence of TB above 40 cases per 100,000; (b) >18 years of age; and (c) capable of giving informed consent.

At the New Patient Health Check all eligible patients were given time to read the participant information sheet (available in Arabic, English, Farsi, Gujarati, Polish, Somali). Telephone interpreters were available on request. Of those who agreed to participate, written informed consent was obtained. Participants were then offered the blood test for latent TB, HIV, hepatitis B and C and blood samples taken from participants. The tests used were as follows: an interferon gamma release assay for TB (QuantiFERON-TB [Quagen, Cellestis, Australia]), an HIV screening assay and HIV confirmatory assay (HIV combo assay and HIV Duo ultra, Abbott Architect and Biomerieux Vidas), a hepatitis B Surface Antigen test (Qualitative II Ultra, Abbott Architect, Biomerieux Vidas), and a Hepatitis C antibody test (Anti HCV, Abbott Architect). Blood samples were paced in labelled study bags and couriered daily to the local laboratory. We provided the practices with the QuantiFERON-TB Gold In-Tubes. Routine care pathways were used for laboratory testing, communicating results, and for referral to follow-up care in specialist services.

Prior to starting data collection, GP baseline data (demographical data and baseline disease rates) were extracted through electronic searches of the INPS-Vision system. Study data on eligible patients were anonymously extracted from patient records every 2 weeks by the healthcare assistant or practice nurse and shared with the research team in an anonymous format. Data were analysed using STATA 12 (StataCorp). To avoid screening bias, we compared demographical differences between patients who accepted and declined the intervention. The study was approved by Bromley Research Ethics Committee, London, and follows STROBE guidelines [21].

## Results

**Figure 1:**
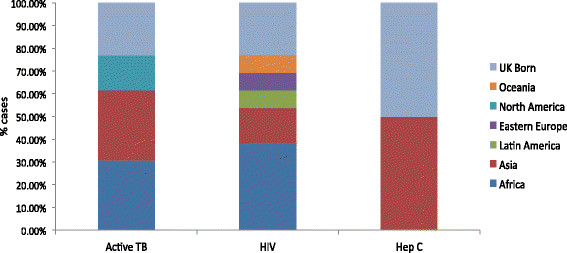
**TB, HIV, and hepatitis C cases according to patient’s region of origin in the general practices at baseline.**

1618 of 5103 (31.7%) patients permanently registered at the Practices were recorded as foreign-born/migrants prior to the study starting; 2552 of 5103 (50.0%) were reported as from an ethnic minority group. Foreign-born patients were significantly under-represented in comparison to 42.8% identified in 2011 Census data [19] reporting the number of foreign-born people living in the surrounding area (Chi-square test −0.111 [95% CI: −0.125 to −0.097]; p < 0.001). From 2009–2012 prior to the study starting, the majority of infectious diseases diagnosed in the permanently registered population was among foreign-born in the two study sites (Figure 1). This group had 10 cases of active TB (13 cases total across the Practice) and 10 cases of HIV (13 total), giving a prevalence rate of active TB in the foreign-born population of 6.2 cases per 1000 (0.62% [95% CI: 0.30 - 1.13]) and the prevalence rate of HIV in the foreign-born population of 6.2 cases per 1000 (0.62% [95% CI: 0.30 - 1.13]). For hepatitis C, the prevalence rate was 0.6 cases per 1000 (0.06% [95% CI: 0.00 - 0.34]). No hepatitis B or latent TB was recorded as detected on the INPS-Vision system.

**Figure 2:**
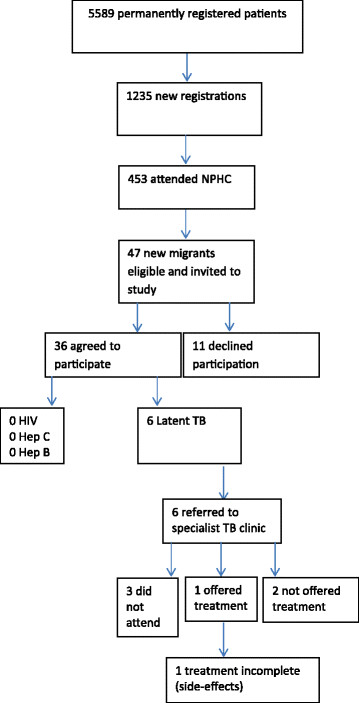
**Flow chart of study.**

During the study period 1235 new patients (both foreign born and UK born) registered permanently, of which 453 (36.7%) attended a New Patient Health Check (Figure 2). 47 of 453 (10.4%) were identified as new migrants representing 22 nationalities (Table 1). 36 of 47 (76.6%) new migrants agreed to participate in the study.

**Table 1:** **Demographic characteristics of new migrants identified in the stud**y

Demographic	All new migrant patients (n = 47)	Agreed to intervention (n = 36)	Declined intervention (n = 11)
**Mean age (range) in years**	32.11 years (18–72)	33.21(18–72)	29.64 (21–37)
**Sex (M/F)**	23/24	17/19	6/5
**Mean years in the UK (range)**	2.28 years (0–10)	2.36 (0–10)	2 (0–6)
**Country of birth**	**Asia (28)** China (6) Hong Kong (1) India (3) Iran (2) Malaysia (11) Pakistan (1) Philippines (1) Thailand (2) Yemen (1)	**Asia (22)** China (4) Hong Kong (1) India (2) Iran (2) Malaysia (9) Pakistan (1) Philippines (1) Thailand (2)	**Asia (6)**
			China (2) India (1) Malaysia (2) Yemen (1)
	**Africa (7)** Libya (1) Morocco (1) Nigeria (2) Somalia (2) South Africa (1)	**Africa (6)** Libya (1) Morocco (1) Nigeria (2) Somalia (2)	**Africa (1)** South Africa (1)
	**Latin America (6)** Argentina (1) Colombia (3) Mexico (1) Peru (1)	**Latin America (3)** Colombia (2) Argentina (1)	**Latin America (3)** Columbia (1) Peru (1) Mexico (1)
	**Eastern Europe (5)** Bulgaria (1) Lithuania (1) Poland (2) Romania (1)	**Eastern Europe (4)** Romania (1) Poland (1) Bulgaria (1) Lithuania (1)	Eastern Europe (1) – Poland (1)
**Requested an interpreter (Yes/No)**	6/41	6/30	0/11

33 of 36 (91.6%) participants were screened using QuantiFERON-TB (vasovagal attack in 1 patient; 1 sample lost in system; 1 not incubated in time), resulting in a positive diagnosis in 6 (16.6%) patients. The prevalence of diagnosed latent TB in new migrants at the survey site was calculated at 181.8 cases per 1000 (18.18% [95% CI 6.98-35.46]). No cases of HIV, hepatitis B, or hepatitis C were found. During the study period, analysis of practice data showed an additional 3 cases of active TB (2 foreign and 1 UK born) and 1 case of HIV (foreign born) diagnosed in patients across the practice who were not included in the study. These diagnoses were made via the patients individual GP, not through our intervention.

Of the 6 patients identified with latent TB, 3 did not attend specialist clinic appointments, 2 were not eligible for chemoprophylaxis under current UK NICE guidelines (they were older than 35 years), and the one patient who was treated suffered an adverse drug reaction prematurely ending therapy. The cost of the screening tests per patient was UK £107.13 (USD$171.39), excluding nurse time and transport costs.

## Discussion and conclusions

Our intervention led to an increase in the prevalence of diagnosed latent TB in comparison to baseline practice data pre-intervention; however, none of these individuals ultimately completed treatment. New migrants presented to these two GP practices in surprisingly low numbers. New migrants were relatively young, represented 22 diverse nationalities, and had been in the UK for short time-periods (mean 2.28 years). The one-stop approach was feasible in this context and acceptability was high (76.6%).

Despite the diverse backgrounds of presenting migrants, their willingness to take the blood test was high, consistent with previous work reporting that migrants are often proactive about their health [22],[23]. Qualitative research we have carried out with migrant health-care leaders around this survey site concluded that there is a great deal of stigma surrounding infectious diseases within new migrant communities, and barriers to accessing screening [23]. Participants expressed support for a community-focused package of health screening combining all of the diseases into a general health check-up, with the aim of reducing stigma around infectious disease screening [23].

A positive test for latent TB in 16.6% of new migrants tested is comparable with a large multicentre cohort study of IGRA screening in UK migrants [11] and concurs with findings from a cluster randomised controlled trial screening for active TB and latent TB of all attendees at New Patient Health Checks [20]. It is of concern that none of the 6 latent TB cases identified in our study ultimately received any treatment, with 3 (50%) of patients not attending referral appointments at specialist secondary-care services. Although numerous factors may explain low secondary care uptake in migrants, there are growing concerns at the current time in the UK and Europe that migrants increasingly fear approaching chargeable secondary services where they may incur immigration checks and potentially unaffordable health-care costs [24]. Despite the fact that screening and treatment for infectious diseases remains free of charge, the current tendency towards a more restrictive approach to health care access based on financial and other penalties can only have negative implications for the promotion of screening and prevention in newly arrived migrants.

Despite the fact that the survey site was in a high migrant area, new migrants presented in surprisingly low numbers, with foreign-born nationals significantly under-represented in relation to local survey data. This is consistent with other data that suggests migrants are somehow being discouraged from registering with primary-care providers [17]. New migrants with low levels of English may also be less aware of New Patient Health Checks and procedures for GP registration. Our data, therefore, run contrary to current discourse in the UK around the disproportionate impact of migrants on primary-care services [16],[25]. We note, however, that there are limitations to our comparison of numbers of foreign-born patients in the practice, versus those in the community, because these data are based on latest Census data available at the time (from 2011) and migration patterns may have changed when data collection at the Practices began.

The one-stop blood test approach is feasible to do in the primary-care context, with healthcare assistants/practice nurses able to engage new migrants in screening at the New Patient Health Check appointment, facilitate laboratory testing and the follow-up/communication of results, and to record basic data on migration status onto the GP INPS-Vision system. However, further data and cost-effectiveness analyses are needed to better understand how the one-stop blood test model can be best used. Until more emphasis is placed on actively promoting primary-care access for newly arrived migrants, including a proactive approach to encouraging them to register with a GP and attend a New Patient Health Check, General Practice may not be the appropriate place to focus screening interventions for multiple diseases in new migrants. A more successful approach may be to offer one-stop screening in Accident and Emergency Departments in secondary care where migrants may present in large numbers [26], or to facilitate active case-finding in the community linked to a robust system that ensures individuals receive treatment at specialist services.

## Authors’ original submitted files for images

Below are the links to the authors’ original submitted files for images. Authors’ original file for figure 1 Authors’ original file for figure 2
