# Supplementary figures and images for: Screening for latent TB, HIV, and hepatitis B/C in new migrants in a high prevalence area of London, UK: a cross-sectional study
Source: BMC Infect Dis. 2014 Dec 3;14:657. doi: 10.1186/s12879-014-0657-2 (PMC4261901; doi:10.1186/s12879-014-0657-2)

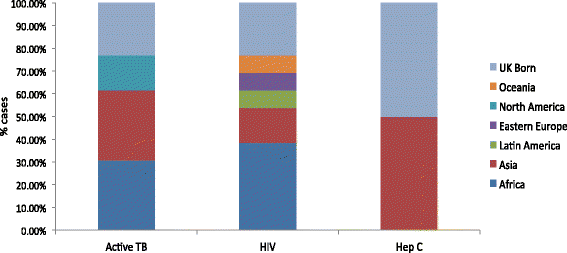

Supplement: Supplementary file 1 — Authors’ original file for figure 1 [file 12879_2014_657_MOESM1_ESM.gif]

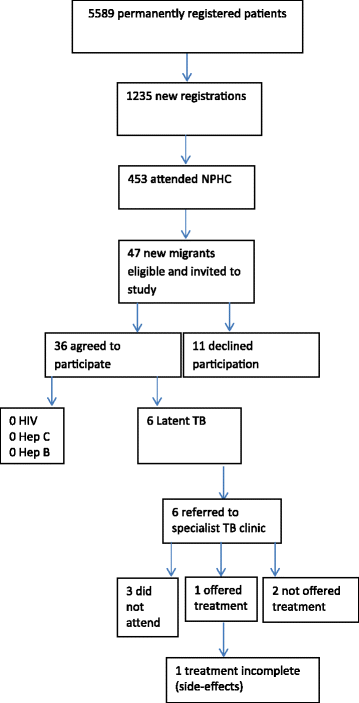

Supplement: Supplementary file 2 — Authors’ original file for figure 2 [file 12879_2014_657_MOESM2_ESM.gif]
